# 3D Chiral Metal Halide Semiconductors

**DOI:** 10.1021/acsenergylett.5c00576

**Published:** 2025-05-22

**Authors:** Marco Moroni, Luca Gregori, Clarissa Coccia, Massimo Boiocchi, Marta Morana, Doretta Capsoni, Andrea Olivati, Antonella Treglia, Giulia Folpini, Maddalena Patrini, Isabel Goncalves, Heyong Wang, Chiara Milanese, Annamaria Petrozza, Edoardo Mosconi, Filippo De Angelis, Lorenzo Malavasi

**Affiliations:** † 19001 University of Pavia, Department of Chemistry and INSTM, Via Taramelli 16, 27100 Pavia, Italy; ‡ University of Perugia, Department of Chemistry, Biology and Biotechnology, Via Elce di Sotto 8, 06123 Perugia, Italy; § University of Pavia, Centro Grandi Strumenti, Via Bassi 21, 27100, Pavia, Italy; ∥ Dipartimento di Scienze della Terra, Università di Firenze, Via La Pira 4, 50121 Florence, Italy; ⊥ 403543 Center for Nano Science and Technology@PoliMi, Istituto Italiano di Tecnologia 20134 Milan, Italy; # Physics Department, Politecnico di Milano, Piazza L. da Vinci, 32, 20133 Milano, Italy; ◧ Istituto di Fotonica e Nanotecnologie, CNR-IFN, 20133 Milan, Italy; □ University of Pavia, Department of Physics, Via Bassi 6, 27100 Pavia, Italy; ● Computational Laboratory for Hybrid/Organic Photovoltaics (CLHYO), Istituto CNR di Scienze e Tecnologie Chimiche “Giulio Natta” (CNR-SCITEC), Via Elce di Sotto 8, 06123 Perugia, Italy; ○ SKKU Institute of Energy Science and Technology (SIEST), Sungkyunkwan University, Suwon 440-746, South Korea

## Abstract

Chiral metal halides are promising materials for nonlinear optics and spin-selective devices. Typically, chirality is introduced via large chiral organic cations, leading to low-dimensional structures and limitations in charge transport. Here, we design a family of chiral metal halides based on the relatively small ditopic *R*/*S*-3-aminoquinuclidine (3-AQ) cation, forming an (*R*/*S*-3AQ)­Pb_2_Br_6_ structure closely related to the 3D corner-sharing octahedral network of perovskites. The resulting material exhibits a direct bandgap, isotropic band structure, and fully 3D photoexcitation. Circular dichroism confirms a chiral anisotropy factor consistent with theoretical predictions. Moreover, the material displays a Rashba effect in the conduction band, which is attributed to spin–orbit coupling and the lack of inversion symmetry. Offering rich chemical tunability and efficient 3D charge transport, this new class of chiral semiconductors provides a promising platform for advancing nonlinear optoelectronic and spintronic devices.

## Graphical abstract

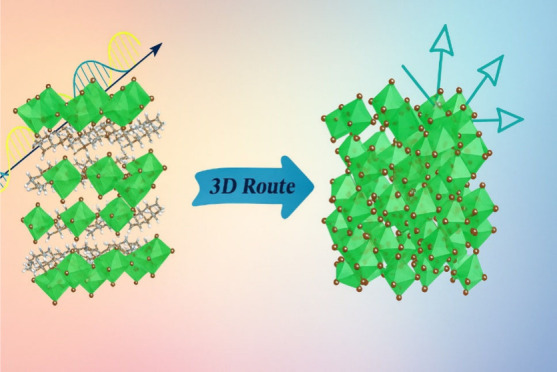

Metal halide perovskites constitute a class of wondrous materials, characterized by huge structural diversity associated with variations in both the inorganic (metal, halide) and organic (A-site cations) sublattices. Besides their success in solar cells and LEDs, such chemical richness can be exploited to engineer a variety of chiral compounds with associated spin-dependent chiroptical electronic properties.^1–8^ Potential technological applications of chiral perovskites range from circularly polarized light emission and detection, chiral sensing, and chiral-induced spin selectivity up to enantioselective synthesis and photocatalysis.^9–11^ Thus, the broad tunability of these materials arises from the interplay of organic and inorganic moieties through the chirality transfer mechanism, whereby chiral A-site organic cations impart chiroptical response to the optically active inorganic framework.^12^ The family of chiral 2D perovskites and low-dimensional metal halides (0D and 1D systems), first reported in 2003,^13,14^ has grown impressively by exploring various organic cations and different metals.^15–21^ These low-dimensional structures were demonstrated to exhibit chiroptical properties as well as spin-polarized absorption, spin-polarized photoluminescence, and second harmonic generation.^22–25^ The inherent carrier and excitonic confinement of such low-dimensional materials and the associated anisotropic transport remain a challenge for their effective integration in devices.

The synthesis of 3D chiral perovskites would represent a significant advancement, lifting the above-mentioned limitations. It is now clear, however, that only small organic cations, such as methylammonium (CH_3_NH_3_^+^) or formamidinium (NH_2_CHNH_2_^+^)), can give rise to 3D perovskites which clearly have no chiral-templating nature.^26,27^ A different approach was proposed by Chen and co-workers through the growth of MAPbBr_3_ single crystals with the addition of micro- or nanoparticles as nucleating agents.^28^ Chirality, in this case, emerges from chiral orientation patterns of the incorporated A-site cations through the formation of chiral supercells, inducing chiroptical activity. The heterogeneous chiral nucleation proposed, however, assures only a partial enantiomeric excess (i.e., the ratio of the two chiral enantiomers) so that control over the handedness (*R*/*L*) selectivity remains a major isssue. In addition, this synthetic process is limited to single crystals whereby thin films are the optimal choice for their embodiment in devices.^28^

Here we face the challenge of achieving 3D chiral metal halide materials by making use of enantiopure cage-like ditopic amines, specifically exploiting *R*/*S*-3-aminoquinuclidine (3-AQ) (Figure 1a). The peculiar steric hindrance of this amine and its relatively small size allow the formation of a 3D chiral metal halide of formula (*R*/*S*-3AQ)­Pb_2_Br_6_ composed exclusively of PbBr_6_ corner-sharing octahedra, similar to the 3D connectivity of prototypical perovskite structures. The new compound presents a direct band gap with chiroptical properties in the circular dichroism response while clearly maintaining signatures typical of 3D semiconductors, as shown by its band dispersion and reduced exciton binding energy and band gap compared to its 2D perovskite counterpart, (*R*/*S*-3-AQ)_2_PbBr_4_·2Br. In line with such properties coupled to the noncentrosymmetric crystal structure, the 3D (*R*/*S*-3AQ)­Pb_2_Br_6_ chiral system is predicted to show a significant Rashba conduction band splitting that makes it potentially amenable for spintronics.^29^

**Figure 1:**
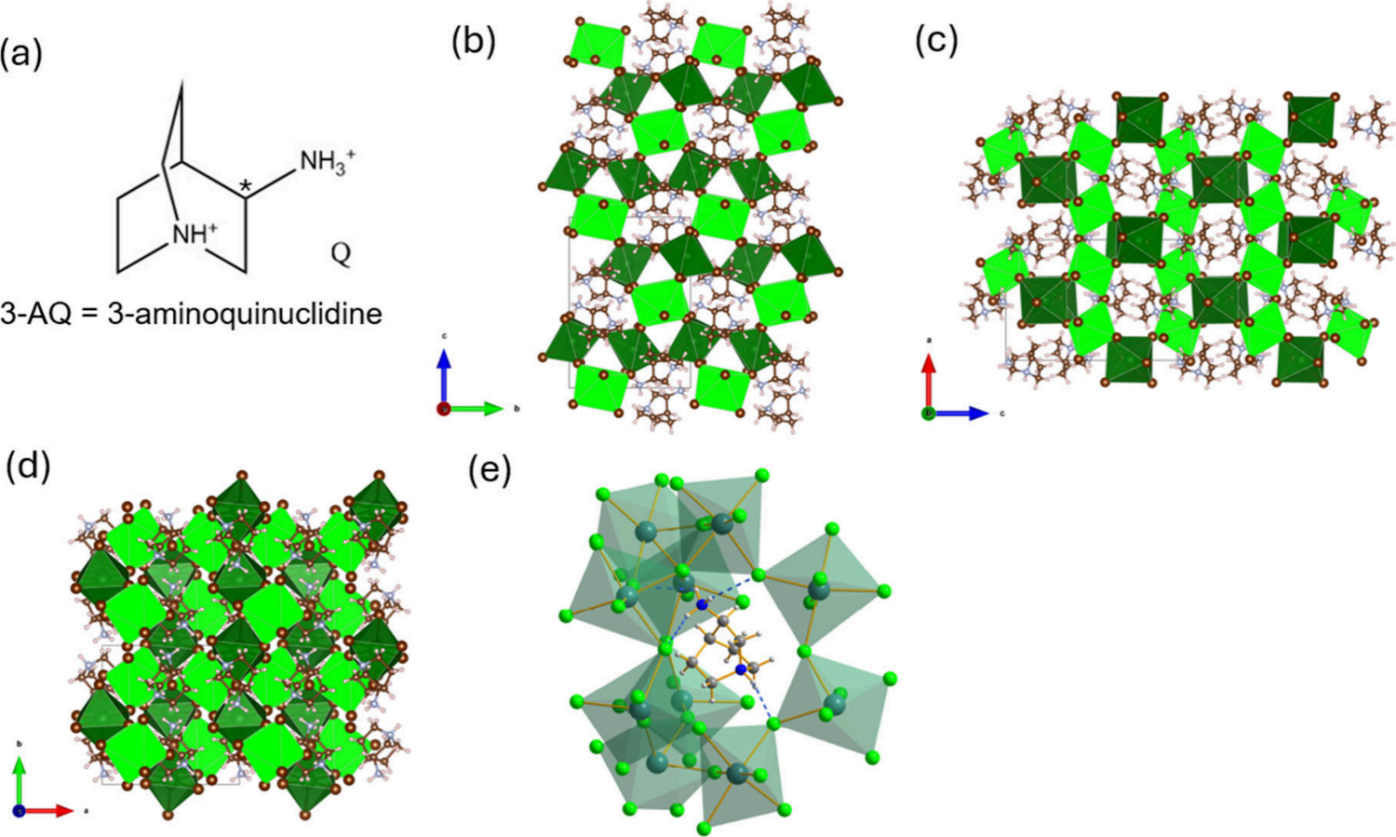
(a) Chemical formula of 3-aminoquinuclidine (3-AQ). The asterisk marks the chiral center. (b–d) Sketches of the crystal structures of (*R*/*S*-3-AQ)­Pb_2_Br_6_ along the *a*, *c*, and *b* axes, respectively. The two crystallographically independent Pb ions are represented with their coordination octahedra in dark green (Pb1) and light green (Pb2), respectively. (e) Highlight of the (*R*-3-AQ)­Pb_2_Br_6_ N–H···Br hydrogen bonds interactions, represented as blue dashed lines.

Samples of (*R*/*S*-3AQ)­Pb_2_Br_6_ and of (*R*/*S*-3-AQ)_2_PbBr_4_·2Br have been synthesized as reported in Methods in the Supporting Information (SI). For (*R*-3AQ)­Pb_2_Br_6_ we grow high-quality single crystals which were used to solve the crystal structure by X-ray diffraction, and the main crystallographic data are reported in Table 1. For the *S*-enantiomer, however, this was hardly possible, and therefore, the crystal structure was Rietveld-refined starting from the single-crystal data of the *R*-enantiomer.

**Table 1:** Crystal Structure Data for [(*R*/*S*-3AQ)_2_PbBr_4_]·2Br and (*R*/*S*-3AQ)­Pb_2_Br_6_ ^a^

	[(***R***-3AQ)_2_PbBr_4_]·2Br	[(***S***-3AQ)_2_PbBr_4_]·2Br	(***R***-3AQ)Pb_2_Br_6_	(***S***-3AQ)Pb_2_Br_6_
**Empirical formula**	C_14_H_32_N_4_Br_6_Pb	C_14_H_32_N_4_Br_6_Pb	C_7_H_16_N_2_Br_6_Pb_2_	C_7_H_16_N_2_Br_6_Pb_2_
**Formula weight**	943.05	943.05	1022.04	1022.04
**Temperature (K)**	298	298	298	298
**Wavelength (Å)**	0.7107	0.7107	0.7107	1.5406
**Crystal system**	Tetragonal	Tetragonal	Orthorhombic	Orthorhombic
**Space group**	*P*4_1_2_1_2	*P*4_3_2_1_2	*P*2_1_2_1_2_1_	*P*2_1_2_1_2_1_
**Lattice parameters (Å)**	*a* = 6.58258(8)	*a* = 6.58253(5)	*a* = 10.9133(2)	*a* = 10.9184(2)
	*b* = 6.58258(8)	*b* = 6.58253(5)	*b* = 11.0771(2)	*b* = 11.0820(2)
	*c* = 56.6268(14)	*c* = 56.6576(8)	*c* = 15.5426(3)	*c* = 15.5470(3)
**Lattice Volume (Å** ^**3**^ **)**	2453.66(9)	2454.96(5)	1878.91(6)	1881.14(6)
**Z**	4	4	4	4
**CCDC code**	2416647	2416648	2416219	–

a For (*S*-3AQ)­Pb_2_Br_6_ we report the Rietveld refinement of powder diffraction pattern.

(*R*/*S*-3AQ)­Pb_2_Br_6_ crystallizes in the orthorhombic chiral space group *P*2_1_2_1_2_1_ (No. 19) at 293 K, providing therefore a noncentrosymmetric structure. The crystal structure of (*R*/*S*-3AQ)­Pb_2_Br_6_ is peculiar and, to our knowledge, not yet reported in the literature for any other metal halide: it is composed of two independent Pb sites (Pb1 and Pb2) forming two distinct types of octahedra with Br ions (see Figure 1b–d). Notably, the reported crystal structure presents an AB_2_X_6_ chemical formula analogous to that of typical perovskitoids.

From Figure 1 one may notice the peculiar arrangement of the PbBr_6_ octahedra: [Pb1Br_6_] octahedra are corner-sharing arranged along the *b*-direction while [Pb2Br_6_] octahedra form an analogous corner-sharing pattern along the *a*-direction. These two perpendicular rows are then again corner-sharing bonded along the *c*-direction creating central voids along the *a* and *b* directions, which host the 3-AQ moieties. N–H···Br hydrogen bond interactions are established for both N atoms of 3-AQ, namely, the tertiary amine of the quinuclidine ring and the primary amine in position 3 of the ring, with H···Br distances in the 2.36–2.99 Å range (Figure 1e).

The PbBr_6_ octahedra present six different Pb–Br bond-lengths; these are, however, very close to each other for both [Pb1Br_6_] and [Pb2Br_6_], lying in the range of 2.93–3.09 Å. Interestingly, the average bond length for [Pb1Br_6_] is 3.0103 Å and 3.0077 Å for [Pb2Br_6_] octahedra which are only slightly longer than the Pb–Br bond length found in cubic MAPbBr_3_ (2.964 Å).^30^ Indeed, the Robinson distortion parameter, *D*,^31^ is very low for both octahedra types, 0.019, while the bond angle variance (σ^2^) is 42.7 and 51.9 deg^2^ for [Pb1Br_6_] and [Pb2Br_6_], respectively (see the list of structural parameters in Table S1, Supporting Information).

To note the difference between the (*R*-3-AQ)­Pb_2_Br_6_ 3D compound and the typical chiral 2D perovskites, we synthesized also the (*R*/*S*-3-AQ)_2_PbBr_4_·2Br compound and solved the crystal structure by SC-XRD (Table 1). The crystal symmetry of both *R*/*S* enantiomers is tetragonal and belongs to the two chiral Sohncke space groups *P*4_1_2_1_2 and *P*4_3_2_1_2, for the *R* and *S* sample, respectively, which are noncentrosymmetric and nonpolar space groups. A sketch of the crystal structure for the two samples is reported in Figure S1 (Supporting Information). Both enantiomers present the typical arrangement of Ruddlesden–Popper 2D perovskites characterized by layers of corner-sharing PbBr_6_ octahedra separated by a bilayer of 3-AQ molecules including an additional layer of bromide anions to ensure electroneutrality. The six Pb–Br bond lengths show a significant distribution from ∼2.78 to ∼ 3.81 Å for both samples, with an average bond length of about 3.20 Å (see also Table S1). The octahedra distortion parameters for (*R*-3-AQ)_2_PbBr_4_·2Br and (*S*-3-AQ)_2_PbBr_4_·2Br are *D* = 0.128 and σ^2^ = 40.77 deg^2^; thus, an increased octahedra distortion is found compared to the 3D system (0.019 vs 0.128).

For both systems we also afforded the preparation of the racemic samples in the form of powders. The X-ray diffraction patterns have been indexed and refined showing an analogous structural arrangement as the chiral compounds but with centrosymmetric space groups, namely *Pmma* for (*rac*-3-AQ)_2_PbBr_4_·2Br and *C*2 (*rac*-3-AQ)_2_PbBr_4_·2Br. The refined patterns are reported in Figure S2, while Table S2 lists the corresponding lattice parameters.

Thermal stability of the *R*-samples has been determined by thermogravimetric analysis in the 30–750 °C temperature range and by differential scanning calorimetry (DSC) in the range from −165 to 65 °C. The results, reported in Figure S3, show good stability of the samples up to 300 °C when the first weight loss occurs. Notably, DSC shows the absence of phase transitions in the considered temperature range, at variance with the typical 3D metal-halide perovskites showing the formation of high-temperature cubic phases.^32^ The phase stability of (*R*-3-AQ)_2_PbBr_4_·2Br and (*R*-3-AQ)­Pb_2_Br_6_ has been further explored by collecting XRD patterns of thin films prepared by spin coating (see Methods in the Supporting Information) left in open air in the laboratory environment (*T* ≈ 22 °C, RH ≈ 40%) as a function of time for up to 47 days, showing significant air and moisture stability (see Figure S4).

To gain insight into the electronic structure of the 3D and 2D samples, we have performed density functional theory (DFT) calculations; see the Supporting Information for computational details. The structures were optimized starting from the crystallographic data at the scalar relativistic PBE level, followed by higher-level HSE06 (α = 0.43) calculations including spin–orbit coupling (SOC). Direct band gap values of 3.46 eV for (*R*/*S*-3-AQ)­Pb_2_Br_6_ and 3.82 eV for (*R*/*S*-3-AQ)_2_PbBr_4_·2Br are calculated, at Γ and M points, respectively (see Figure 2 and Table S3).^33–35^

**Figure 2:**
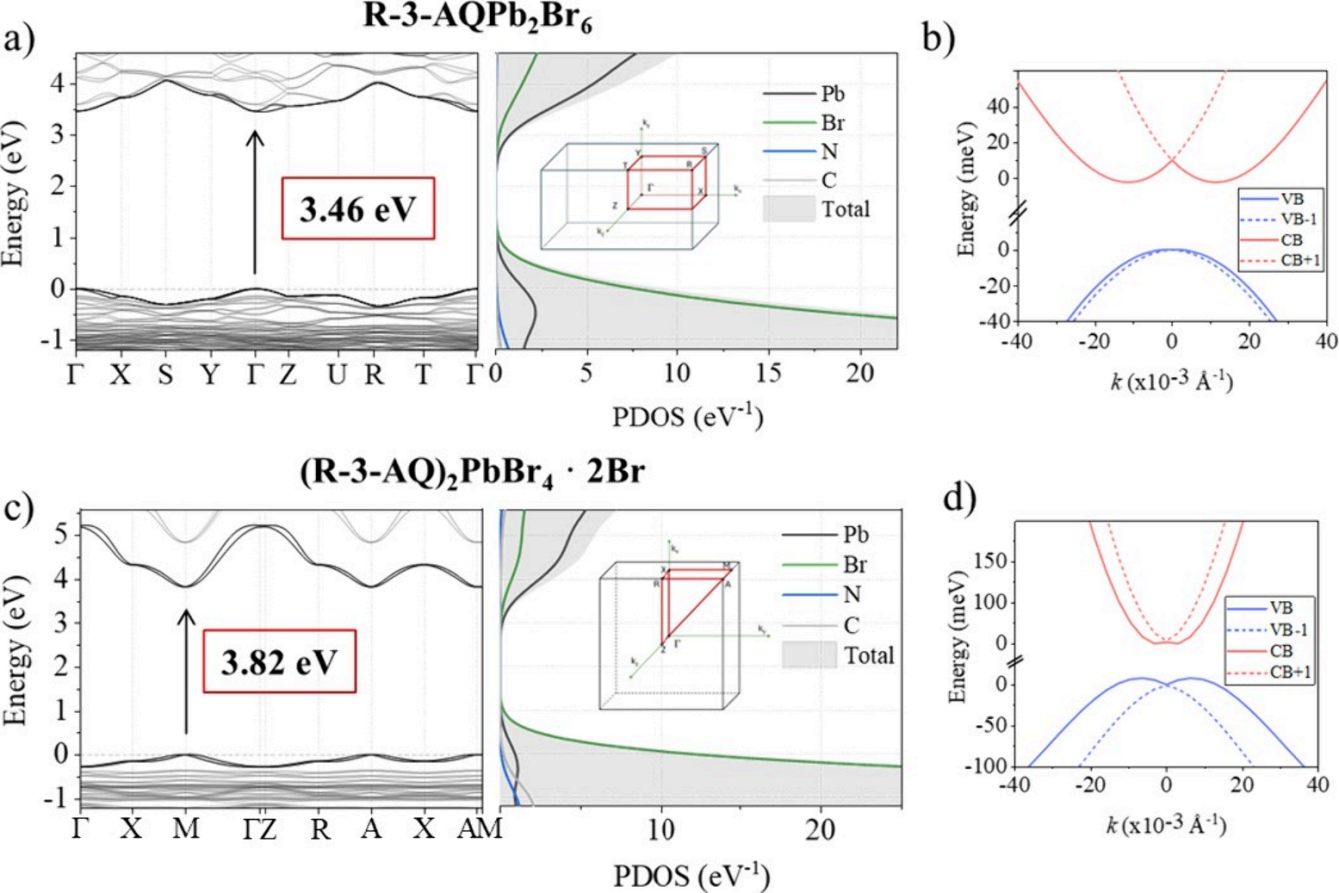
(a, c) Electronic band structures and projected density of states (PDOS) for the 3D and 2D materials, respectively. The PDOS was computed using the HSE06-SOC level of theory, whereas the band structures were initially obtained with PBE-SOC and subsequently corrected to align with the HSE06-SOC band gap. The insets in the PDOS panels illustrate the corresponding Brillouin zone representations. (b, d) Rashba splitting of the valence and conduction bands for the respective materials, highlighting spin–orbit coupling effects. The energy zero reference is set to the valence band maximum (VBM) and conduction band minimum (CBM) to enhance the visualization of the splitting.

The projected density of states (PDOS) of the *R*-isomers, Figure 2a–c, shows that the band edges in both 3D and 2D materials are dominated by Pb and Br contributions, while the C, N, and H atoms from the organic molecules contribute to states far away from the band edges, similar to typical lead-halide perovskites.^36^ The k-point path used for the band structure calculations, shown in the inset in Figure 2, was selected according to standardized high-symmetry paths generated by SeeK-path and validated with the Bilbao Crystallographic Server, ensuring an accurate and complete representation of the electronic structure starting from the crystallographic information obtained from the experiments.^37,38^ Figure 2a–c shows a direct band gap for both structural arrangements. Importantly, the 2D band structure shows a flat band dispersion along the direction perpendicular to the inorganic perovskite layers, i.e., M → A (see Figure S11 in the Supporting Information), while the (*R*/*S*-3-AQ)­Pb_2_Br_6_ system has a clearly isotropic band structure, indicating that the charge carriers can be transported along the 3D octahedra network. Carrier effective masses for (*R*/*S*-3-AQ)­Pb_2_Br_6_ were determined by parabolic fitting along the Γ → Y/Γ → Z in the band gap region, finding *m*_h_* and *m*_e_* values of 0.54/0.66 and 0.34/0.53 *m*_0_, respectively, where *m*_0_ is the electron mass. Effective masses along other crystallographic directions are listed in Table S3. An exciton reduced mass of 0.21/0.29 *m*_0_ is thus estimated. It is interesting to compare calculated effective masses for (*R*/*S*-3-AQ)­Pb_2_Br_6_ to those of typical 3D perovskites, e.g., MAPbBr_3_.^39^ Although the values obtained for our system are a factor ∼1.5 times higher than those for MAPbBr_3_ (*m*_h_* and *m*_e_* are 0.31 and 0.27 m_0_, respectively), a similar trend is observed, with electrons being more mobile than holes.

Notably, both 3D and 2D systems display a Rashba/Dresselhaus spin splitting of the electronic band structure (Figure 2b–d),^40–42^ a characteristic that can be exploited in spintronic applications. The Rashba splitting parameters, including the energy splitting *ε*_*c*/*v*_^≠^, moment offset (Δ*k*), and Rashba coefficient (α), are reported in Tables S4 and S5. For (*R*/*S*-3-AQ)­Pb_2_Br_6_ a significant Rashba effect is observed in the conduction band with α = 2.22 eV Å. In contrast, the valence band shows weaker Rashba splitting, indicating minimal spin–orbit interactions in this band. On the other hand, high values of α were found for the 2D (*R*/*S*-3-AQ)_2_PbBr_4_·2Br (7.12 and 8.19 for the valence and conduction band, respectively) in line with a higher octahedra distortion for the 2D species with respect to the 3D (see Table S1).

Structural and electronic structure calculations confirm a 3D character for the novel (*R*/*S*-3-AQ)­Pb_2_Br_6_ chiral compound. The optical properties of the 3D and 2D systems have thus been measured by UV–vis and PL spectroscopy and by circular dichroism (CD). The absorption measurements at 300 K are shown in Figure 3a,b for the four samples as Tauc plots, while Figures S5 and S6 report a comparison of room-temperature data with those recorded at 77 K. We observe absorption features at 3.39 and 3.64 eV for (*R*/*S*-3-AQ)­Pb_2_Br_6_ and for (*R*/*S*-3-AQ)_2_PbBr_4_·2Br, respectively, compatible with excitonic absorption. The band gap, as identified by fitting the subsequent absorption rise, is found at 3.72 and 4.05 eV for the 3D and 2D structures, respectively. Both data are in good agreement with calculated values; in particular, the band gap increases moving from 3D to 2D structures (0.33 vs 0.36 eV). Thus, for both the excitonic peak and band gap, a noticeable blue shift is observed when moving to the 2D structural arrangement, consistent with a more delocalized excited state and band dispersion in the 3D case. For the (*R*/*S*-3-AQ)­Pb_2_Br_6_, the exciton binding energy, *E*_b_, is 480 meV, consistent with the presence of a stable excitonic population at room temperature (Figure 3a). For (*R*/*S*-3-AQ)_2_PbBr_4_·2Br, the exciton binding energy is 500–540 meV for the *R* and *S* enantiomers, respectively, consistent with a higher degree of confinement in the 2D arrangement.

**Figure 3:**
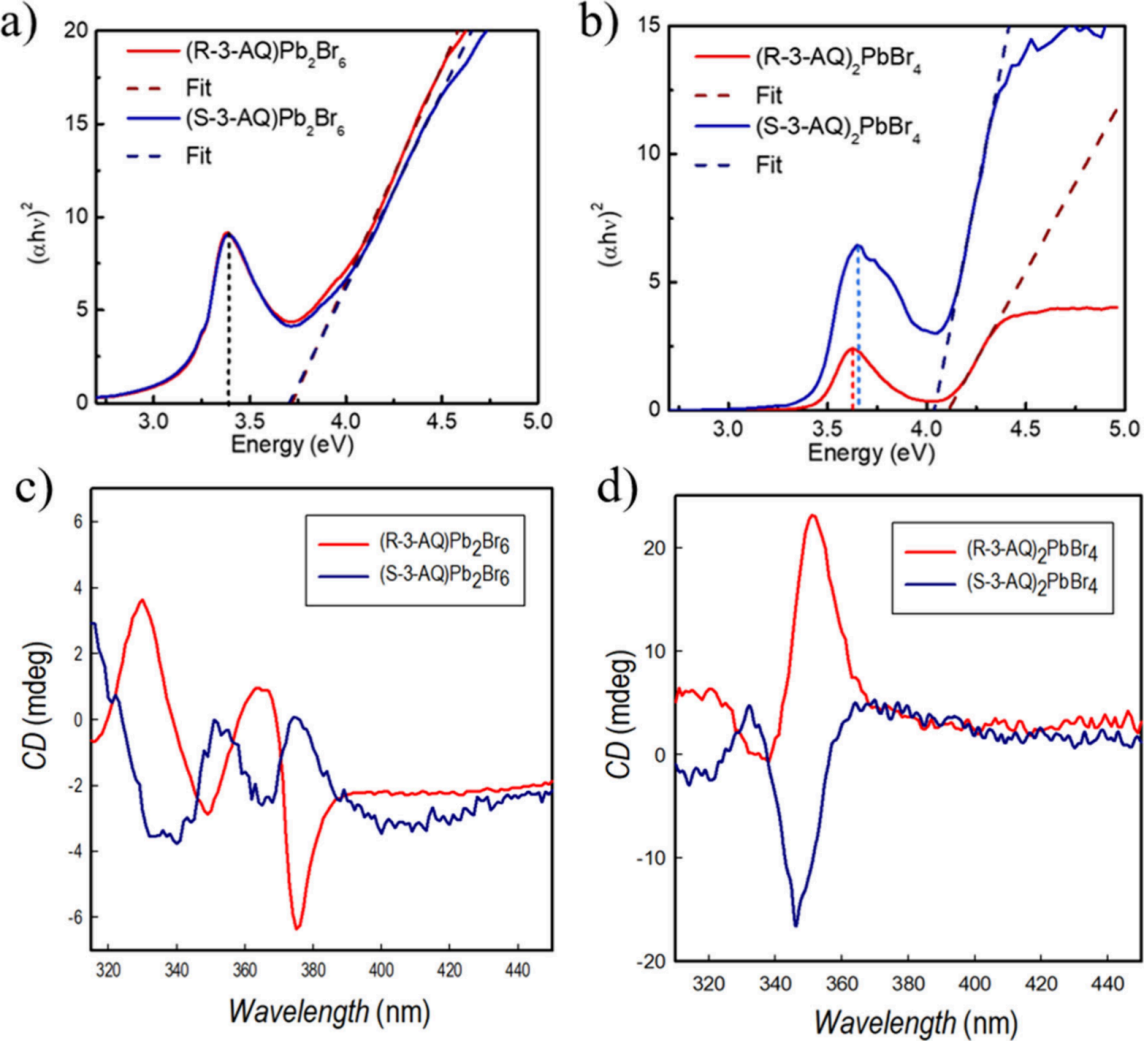
Tauc plot for (a) (*R*/*S*-3AQ)­Pb_2_Br_6_ and (b) (*R*/*S*-3-AQ)_2_PbBr_4_·2Br at 300 K; CD spectra for (c) (*R*/*S*-3AQ)­Pb_2_Br_6_ and (d) (*R*/*S*-3-AQ)_2_PbBr_4_·2Br.

The PL emission of (*R*/*S*-3AQ)­Pb_2_Br_6_, Figure S7, is centered at 1.7 eV, with a significant Stokes shift and a bandwidth of about 500 meV. For the 2D system the photoluminescence spectra are even more broadband, peaking at about 2.3 eV, with a bandwidth up to 800 meV, consistent with the high distortion of the octahedral layers (see Figure S7 and Table S1).

Lastly, we characterized the CD response of thin films of 300 nm average thickness; see Methods in the Supporting Information. The XRD patterns of the thin films are shown in Figure S8, indicating a single-phase nature. Their morphology has been investigated by atomic force microscopy, and representative images are reported in Figure S9. The CD spectra of (*R*/*S*-3-AQ)­Pb_2_Br_6_, Figure 3c, present distinct peaks in the range of 315–400 nm, with the one centered at a higher wavelength corresponding to the absorption edge. The presence of an opposite sign in the CD peaks for the *R* and *S* samples confirms the chirality transfer from the ditopic 3-AQ chiral cation to the Pb–Br inorganic framework. The chiral anisotropy factor, *g*_CD_, has been calculated according to the following equation:

1 $$g_{\mathrm{CD}}=\frac{A_{L}-A_{R}}{2\left(A_{L}+A_{R}\right)}$$

where *A*_L_ and *A*_R_ are the absorbance of the sample for left- and right-handed circularly polarized light, respectively, and resulted in 6 × 10^–5^ for (*R*-3-AQ)­Pb_2_Br_6_ and −9 × 10^–5^ for (*S*-3-AQ)­Pb_2_Br_6_. The CD spectra for the 2D perovskites are shown in Figure 3d. A single peak with opposite sign around 345–350 nm (again in line with the absorption edge, cfr. Figure S6) can be observed, with a slight blue-shift with respect to the 3D systems. *g*_CD_ values are 4 × 10^–4^ for (*R*-3-AQ)_2_PbBr_4_·2Br and −3 × 10^–4^ for (*S*-3-AQ)_2_PbBr_4_·2Br. Interestingly, the chiral anisotropy factor is 1 order of magnitude larger for the 2D perovskite with respect to the 3D compound. Such a result further supports the correlation between the structural dimensionality and *g*_CD_ values. Indeed, it has been recently shown that there is a progressive reduction of roughly 1 order of magnitude of the anisotropy factor passing from 0D to 1D to 2D chiral systems.^15,43,44^ Thus, the first chiral 3D system characterized by only corner-sharing octahedra allows extending the correlation, further confirming such a progressive reduction which seems closely related to the higher amount of chiral ligand in lower-dimensional systems and the reduced lattice rigidity.^45,46^ CPL measurements at 77 K (Figure S10) further confirm the trend of dimensionality also on emission properties. The 2D system has a detectable CPL with *g*_CPL_ of about 0.2%, while in contrast, the 3D system does not show appreciable CPL.

In summary, a new chiral metal halide semiconductor with a 3D structure, namely, (*R*/*S*-3-AQ)­Pb_2_Br_6_, has been synthesized by using a small and sterically hindered ditopic cation, providing the first chiral halide structural network composed of corner-shared octahedra only. The calculation of the electronic band structure for (*R*/*S*-3-AQ)­Pb_2_Br_6_ demonstrates an improved isotropic carrier mobility with respect to the (*R*/*S*-3-AQ)_2_PbBr_4_·2Br 2D system, which shows transport confined in the plane of the inorganic layers. These findings are corroborated by the photophysical properties of the semiconductor which shows a lower degree of localization of the photoexcitation, and of excitation nature, with respect to its 2D counterpart. The novel 3D system reported has a clear chiroptical response in the CD spectra which scales with the structural dimensionality and shows a sizable Rashba splitting in the conduction band.

Overall, this novel family of chiral 3D hybrid metal halide semiconductors has the potential to overcome the limitation of anisotropic transport of actual chiral systems, paving the way for the development of chiroptical and spintronic devices. Moreover, the well-recognized properties of tunability of hybrid metal halides through chemical alloying could be applied to (*R*/*S*-3-AQ)­Pb_2_Br_6_ to further manipulate its chiroptical and transport properties, thus creating a library of novel chiral 3D systems.

## Supplementary Material



## Notes

Methods; structural and computational details; and additional figures presenting structural, thermal, optical, and morphological properties (PDF)

## References

[1] Dang Y., Liu X., Cao B., Tao X. (2021). Chiral Halide Perovskite Crystals
for Optoelectronic Applications. Matter.

[2] Long G., Sabatini R., Saidaminov M. I., Lakhwani G., Rasmita A., Liu X., Sargent E. H., Gao W. (2020). Chiral-Perovskite Optoelectronics. Nat. Rev. Mater..

[3] Malavasi, L. ; Moroni, M. ; Coccia, C. Chiral Metal Halide Perovskites: Focus on Lead-Free Materials and Structure-Property Correlations; preprint; Chemistry and Materials Science, 2023. 10.20944/preprints202307.0836.v1. PMC10457802 37630418

[4] Min J., Choi Y., Kim D., Park T. (2024). Beyond Imperfections:
Exploring Defects for Breakthroughs in Perovskite Solar Cell Research. Adv. Energy Mater..

[5] Wang H., Treglia A., Albaqami M. D., Gao F., Petrozza A. (2024). Tin-Halide
Perovskites for Near-Infrared Light-Emitting Diodes. ACS Energy Lett..

[6] Yuan F., Folpini G., Liu T., Singh U., Treglia A., Lim J. W. M., Klarbring J., Simak S. I., Abrikosov I. A., Sum T. C., Petrozza A., Gao F. (2024). Bright and Stable Near-Infrared
Lead-Free Perovskite Light-Emitting Diodes. Nat. Photonics.

[7] Luo X., Liu X., Lin X., Wu T., Wang Y., Han Q., Wu Y., Segawa H., Han L. (2024). Recent Advances of
Inverted Perovskite
Solar Cells. ACS Energy Lett..

[8] Xing S., Yuan Y., Zhang G., Zhang S., Lian Y., Tang W., Zhou K., Liu S., Gao Y., Ren Z., Zhang G., Sun T., Zhao B., Di D. (2024). Energy-Efficient
Perovskite LEDs with Rec. 2020 Compliance. ACS
Energy Lett..

[9] Chen C., Gao L., Gao W., Ge C., Du X., Li Z., Yang Y., Niu G., Tang J. (2019). Circularly
Polarized
Light Detection Using Chiral Hybrid Perovskite. Nat. Commun..

[10] Lee C. U., Lee H., Jeong C.-S., Ma S., Jang G., Park Y. S., Yun J., Lee J., Son J., Jeong W., Yang S., Park J. H., Woo K., Moon J. (2024). Enhanced Stability
of Spin-Dependent Chiral 2D Perovskite Embedded PV-Biased Anode via
Cross-Linking Strategy. ACS Energy Lett..

[11] Chen D., Tang B., Sergeev A. A., Wu Y., Liu H., Zhu D., Hu S., Wong K. S., Yip H.-L., Rogach A. L. (2025). Green Spin
Light-Emitting Diodes Enabled by Perovskite Nanocrystals *in
Situ* Modified with Chiral Ligands. ACS Energy Lett..

[12] Ma S., Ahn J., Moon J. (2021). Chiral Perovskites for Next-Generation
Photonics: From
Chirality Transfer to Chiroptical Activity. Adv. Mater..

[13] Billing D. G., Lemmerer A. (2003). Bis­[(*S*)-β-Phenethylammonium]
Tribromoplumbate­(II). Acta Crystallogr. E Struct
Rep. Online.

[14] Billing D. G., Lemmerer A. (2007). Synthesis, Characterization and Phase
Transitions in
the Inorganic-Organic Layered Perovskite-Type Hybrids [(C _
*n*
_ H _2 *n*+1_ NH _3_) _2_ PbI _4_], *n* = 4,
5 and 6. Acta Crystallogr. B Struct Sci..

[15] Coccia C., Morana M., Mahata A., Kaiser W., Moroni M., Albini B., Galinetto P., Folpini G., Milanese C., Porta A., Mosconi E., Petrozza A., De Angelis F., Malavasi L. (2024). Ligand-Induced Chirality in ClMBA _2_ SnI _4_ 2D Perovskite**. Angew. Chem. Int.
Ed.

[16] Wang H., Li J., Lu H., Gull S., Shao T., Zhang Y., He T., Chen Y., He T., Long G. (2023). Chiral Hybrid Germanium­(II)
Halide with Strong Nonlinear Chiroptical Properties. Angew. Chem. Int. Ed.

[17] Asensio Y., Bahmani Jalali H., Marras S., Gobbi M., Casanova F., Mateo-Alonso A., Di Stasio F., Rivilla I., Hueso L. E., Martín-García B. (2024). Circularly
Polarized Photoluminescence
in Chiral Hybrid Organic-Inorganic Manganese Halide Perovskites: From
Bulk Materials to Exfoliated Flakes. Advanced
Optical Materials.

[18] Dibenedetto A., Coccia C., Boiocchi M., Moroni M., Milanese C., Malavasi L. (2024). Synthesis and Characterization of Cu-Containing Chiral
Metal Halides and Role of Halogenation of the Organic Ligand. J. Phys. Chem. C.

[19] Hao J., Lu H., Mao L., Chen X., Beard M. C., Blackburn J. L. (2021). Direct
Detection of Circularly Polarized Light Using Chiral Copper Chloride-Carbon
Nanotube Heterostructures. ACS Nano.

[20] Peng H., Liu Q., Lu Y.-Z., Yang S.-J., Qi J.-C., Chen X.-G., Liao W.-Q. (2023). A Chiral Two-Dimensional Perovskite-like Lead-Free
Bismuth­(iii) Iodide Hybrid with High Phase Transition Temperature. Chem. Commun..

[21] Wu P., Pietropaolo A., Fortino M., Bando M., Maeda K., Nishimura T., Shimoda S., Sato H., Naga N., Nakano T. (2023). Amplified
Chirality Transfer to Aromatic Molecules
through Non-specific Inclusion by Amorphous, Hyperbranched Poly­(Fluorenevinylene)
Derivatives. Angew. Chem. Int. Ed.

[22] Moroni M., Coccia C., Malavasi L. (2024). Chiral 2D
and Quasi-2D Hybrid Organic
Inorganic Perovskites: From Fundamentals to Applications. Chem. Commun..

[23] Long G., Jiang C., Sabatini R., Yang Z., Wei M., Quan L. N., Liang Q., Rasmita A., Askerka M., Walters G., Gong X., Xing J., Wen X., Quintero-Bermudez R., Yuan H., Xing G., Wang X. R., Song D., Voznyy O., Zhang M., Hoogland S., Gao W., Xiong Q., Sargent E. H. (2018). Spin Control in Reduced-Dimensional
Chiral Perovskites. Nature Photon.

[24] Ma J., Fang C., Chen C., Jin L., Wang J., Wang S., Tang J., Li D. (2019). Chiral 2D
Perovskites
with a High Degree of Circularly Polarized Photoluminescence. ACS Nano.

[25] Li Z., Hong E., Zhang X., Deng M., Fang X. (2022). Perovskite-Type
2D Materials for High-Performance Photodetectors. J. Phys. Chem. Lett..

[26] Saparov B., Mitzi D. B. (2016). Organic-Inorganic
Perovskites: Structural Versatility
for Functional Materials Design. Chem. Rev..

[27] Mao L., Stoumpos C. C., Kanatzidis M. G. (2019). Two-Dimensional Hybrid Halide Perovskites:
Principles and Promises. J. Am. Chem. Soc..

[28] Chen G., Liu X., An J., Wang S., Zhao X., Gu Z., Yuan C., Xu X., Bao J., Hu H.-S., Li J., Wang X. (2023). Nucleation-Mediated Growth of Chiral 3D Organic-Inorganic
Perovskite Single Crystals. Nat. Chem..

[29] Lesne E., Fu Y., Oyarzun S., Rojas-Sánchez J. C., Vaz D. C., Naganuma H., Sicoli G., Attané J.-P., Jamet M., Jacquet E., George J.-M., Barthélémy A., Jaffrès H., Fert A., Bibes M., Vila L. (2016). Highly Efficient
and Tunable Spin-to-Charge Conversion through Rashba Coupling at Oxide
Interfaces. Nat. Mater..

[30] Liang A., Gonzalez-Platas J., Turnbull R., Popescu C., Fernandez-Guillen I., Abargues R., Boix P. P., Shi L.-T., Errandonea D. (2022). Reassigning
the Pressure-Induced Phase Transitions of Methylammonium Lead Bromide
Perovskite. J. Am. Chem. Soc..

[31] Robinson K., Gibbs G. V., Ribbe P. H. (1971). Quadratic Elongation: A Quantitative
Measure of Distortion in Coordination Polyhedra. Science.

[32] Poglitsch A., Weber D. (1987). Dynamic Disorder in Methylammoniumtrihalogenoplumbates
(II) Observed
by Millimeter-Wave Spectroscopy. J. Chem. Phys..

[33] Gregori L., Meggiolaro D., De Angelis F. (2024). Quantifying the Effect of Interfacial
Dipoles on the Energy Level Alignment of Metal-Halide Perovskites. ACS Energy Lett..

[34] Marchal N., Mosconi E., García-Espejo G., Almutairi T. M., Quarti C., Beljonne D., De Angelis F. (2021). Cation Engineering
for Resonant Energy Level Alignment in Two-Dimensional Lead Halide
Perovskites. J. Phys. Chem. Lett..

[35] Meggiolaro D., Mosconi E., Proppe A. H., Quintero-Bermudez R., Kelley S. O., Sargent E. H., De Angelis F. (2019). Energy Level
Tuning at the MAPbI_3_ Perovskite/Contact Interface Using
Chemical Treatment. ACS Energy Lett..

[36] Umari P., Mosconi E., De Angelis F. (2014). Relativistic GW Calculations on CH3NH3PbI3
and CH3NH3SnI3 Perovskites for Solar Cell Applications. Sci. Rep.

[37] Hinuma Y., Pizzi G., Kumagai Y., Oba F., Tanaka I. (2017). Band Structure
Diagram Paths Based on Crystallography. Comput.
Mater. Sci..

[38] Aroyo M. I., Kirov A., Capillas C., Perez-Mato J. M., Wondratschek H. (2006). Bilbao Crystallographic Server. II. Representations
of Crystallographic Point Groups and Space Groups. Acta Crystallogr. A Found Crystallogr..

[39] Mosconi E., Umari P., De Angelis F. (2016). Electronic
and Optical Properties
of MAPbX_3_ Perovskites (X = I, Br, Cl): A Unified DFT and
GW Theoretical Analysis. Phys. Chem. Chem. Phys..

[40] Mosconi E., Etienne T., De Angelis F. (2017). Rashba Band
Splitting in Organohalide
Lead Perovskites: Bulk and Surface Effects. J. Phys. Chem. Lett..

[41] Etienne T., Mosconi E., De Angelis F. (2016). Dynamical
Origin of the Rashba Effect
in Organohalide Lead Perovskites: A Key to Suppressed Carrier Recombination
in Perovskite Solar Cells?. J. Phys. Chem. Lett..

[42] Jana M. K., Song R., Liu H., Khanal D. R., Janke S. M., Zhao R., Liu C., Valy Vardeny Z., Blum V., Mitzi D. B. (2020). Organic-to-Inorganic Structural Chirality
Transfer in a 2D Hybrid Perovskite and Impact on Rashba-Dresselhaus
Spin-Orbit Coupling. Nat. Commun..

[43] Coccia C., Moroni M., Treglia A., Boiocchi M., Yang Y., Milanese C., Morana M., Capsoni D., Porta A., Petrozza A., Stroppa A., Malavasi L. (2024). Unraveling
the Role
of Structural Topology on Chirality Transfer and Chiroptical Properties
in Chiral Germanium Iodides. J. Am. Chem. Soc..

[44] Moroni M., Coccia C., Malavasi L. (2024). Chiral 2D
and Quasi-2D Hybrid Organic
Inorganic Perovskites: From Fundamentals to Applications. Chem. Commun..

[45] Das R., Hossain M., Mahata A., Swain D., De Angelis F., Santra P. K., Sarma D. D. (2023). Unique
Chiro-Optical Properties of
the Weakly-2D (R-/S-MBA) _2_ CuBr _4_ Hybrid Material. ACS Materials Lett..

[46] Zhang Z., Wang Z., Sung H. H.-Y., Williams I. D., Yu Z.-G., Lu H. (2022). Revealing the Intrinsic
Chiroptical Activity in Chiral Metal-Halide
Semiconductors. J. Am. Chem. Soc..

