# Supplementary material for: 3D Chiral Metal Halide Semiconductors
Source: ACS Energy Lett. 2025 May 22;10(6):2906–12. doi: 10.1021/acsenergylett.5c00576 (PMC12172314; doi:10.1021/acsenergylett.5c00576)
Supplement: Supplementary file 1 [file nz5c00576_si_001.pdf]

Supplementary information

3D Chiral Metal Halide Semiconductors

Marco Moroni,[a] *Luca Gregori,*[b,i] *Clarissa Coccia,*[a] *Massimo Boiocchi,*[c] *Marta Morana,*[d] *Doretta Capsoni,*[a] *Andrea Olivati,*[e,f] *Antonella Treglia,*[e] *Giulia Folpini,*[e,g] *Maddalena Patrini,*[h] *Isabel Goncalves,*[e] *Heyong Wang,*[e] *Chiara Milanese,*[a] *Annamaria Petrozza,*[e] *Edoardo Mosconi,*[i] *Filippo De Angelis,*[b,i,l] *and Lorenzo Malavasi,*[a,*]

[a]University of Pavia, Department of Chemistry and INSTM, Via Taramelli 16, 27100, Pavia, Italy.

[b]University of Perugia, Department of Chemistry, Biology and Biotechnology, Via Elce di Sotto 8, 06123, Perugia, Italy.

[c]University of Pavia, Centro Grandi Strumenti, Via Bassi 21, 27100, Pavia, Italy.

[d]Dipartimento di Scienze della Terra, Università di Firenze, Via La Pira 4, 50121 Florence, Italy

[e]Center for Nano Science and Technology@PoliMi, Istituto Italiano di Tecnologia 20134 Milan, Italy.

[f] Physics Department, Politecnico di Milano, Piazza L. da Vinci, 32, 20133 Milano, Italy.

[g] Istituto di Fotonica e Nanotecnologie, CNR-IFN, 20133 Milan, Italy.

[h]University of Pavia, Department of Physics, Via Bassi 6, 27100, Pavia, Italy.

[i]Computational Laboratory for Hybrid/Organic Photovoltaics (CLHYO), Istituto CNR di Scienze e Tecnologie Chimiche "Giulio Natta" (CNR-SCITEC), Via Elce di Sotto 8, 06123 Perugia, Italy

[l]SKKU Institute of Energy Science and Technology (SIEST), Sungkyunkwan University, Suwon 440-746, South Korea

Corresponding Authors

Lorenzo Malavasi, email: lorenzo.malavasi@unipv.it; tel. +39 382 987921; Edoardo Mosconi, edoardo@thch.unipg.it

Table S1. Octahedral distortion parameters and bond lengths for (*R*/*S*-3-AQ)Pb_2Br_6 and (*R*/*S*-3-AQ)$_2PbBr_4$·2Br.

Compound	Polyhedron	Distortion Index (x10^3)	Bond angle variance (deg^2)	Quadratic elongation	Bond lengths (Å)	M-X-M (deg)
**(*R*-3AQ)Pb_2Br_6**	$Pb1Br_6$	19.4	42.7683	1.0127	2.9309(11)<br>2.9568(11)<br>2.9678(13)<br>3.0545(12)<br>3.0618(13)<br>3.0901(11)	120.10(4)<br>130.98(4)<br>132.00(4)<br>136.24(4)<br>136.52(4)<br>146.50(4)
	$Pb2Br_6$	18.9	51.9884	1.0158	2.9349(13)<br>2.9485(13)<br>2.9694(10)<br>3.0298(10)<br>3.0703(14)<br>3.0933(13)	
**(*S*-3AQ)Pb_2Br_6**	$Pb1Br_6$	19.9	50.2638	1.0150	2.939(10)<br>2.95(2)<br>3.00(3)<br>3.085(17)<br>3.085(16)<br>3.086(10)	118.0(5)<br>129.2(5)<br>130.0(5)<br>136.0(4)<br>137.2(7)<br>143.1(7)
	$Pb2Br_6$	14.0	65.4779	1.0201	2.950(19)<br>2.991(15)<br>3.02(3)<br>3.03(3)<br>3.05(3)<br>3.139(16)	
**[(*R*-3AQ)$_2PbBr_4$]·2Br**		129.0	40.6743	1.0350	2.7824(8)<br>3.0003(12)<br>3.8207(8)	170.84(4)
**[(*S*-3AQ)$_2PbBr_4$]·2Br**		128.8	40.7754	1.0349	2.7827(8)<br>3.0024(12)<br>3.8206(8)	170.79(4)

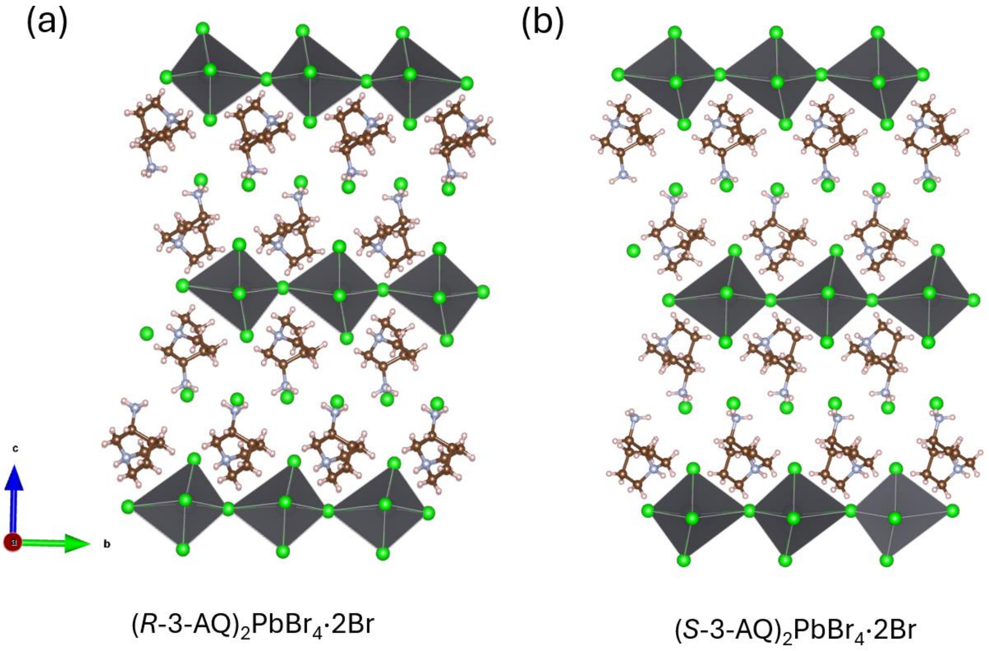

Figure S1. (a-b) Sketches of the crystal structures of (*R*/*S*-3-AQ)$_2$PbBr$_4$·2Br along the *c*-axis; green spheres represent Br ions.

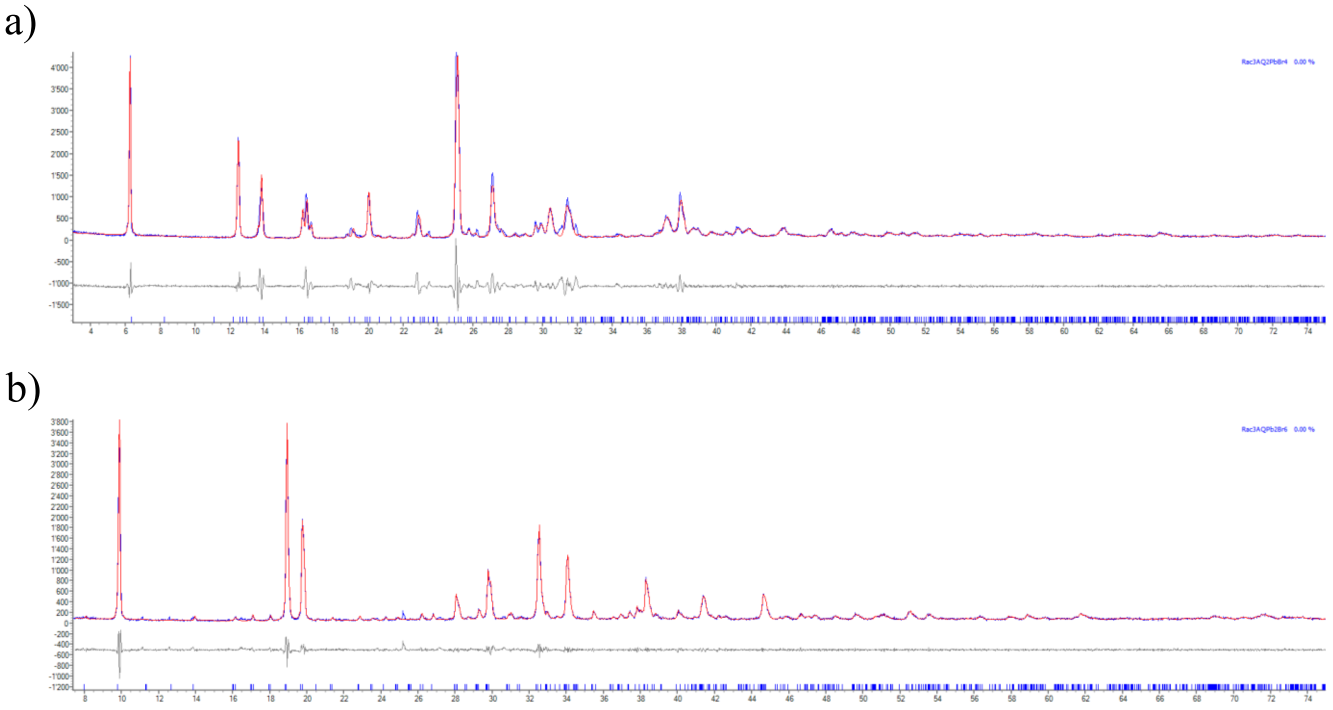

Figure S2. Whole powder pattern Le Bail refinement of (a) (*rac*-3-AQ)$_2$PbBr$_4$·2Br and (b) (*rac*-3-AQ)Pb$_2$Br$_6$ PXRD patterns in terms of experimental, calculated and difference traces (blue, red and grey, respectively). The blue ticks indicate the positions of the Bragg reflections.

Table S2. Crystal structure data for (*rac*-3AQ)$_2$PbBr$_4$·2Br and (*rac*-3AQ)Pb$_2$Br$_6$.

	**(*rac*-3AQ)$_2$PbBr$_4$·2Br**	**(*rac*-3AQ)Pb$_2$Br$_6$**
**Empirical formula**	$C_{14}H_{32}N_4Br_6Pb$	$C_7H_{16}N_2Br_6Pb_2$
**Crystal system**	Monoclinic	Orthorhombic
**Space group**	*C*2	*Pmma*
**Lattice parameters (Å)**	*a* = 27.717(3)	*a* = 10.9730(5)
	b = 11.6081(8)	*b* = 15.6188(6)
	c = 7.9741(6)	*c* = 11.0562(4)
**β Angle (°)**	92.163(6)	-
**Lattice Volume (Å^3)**	2463.163(6)	1894.9(1)

a)

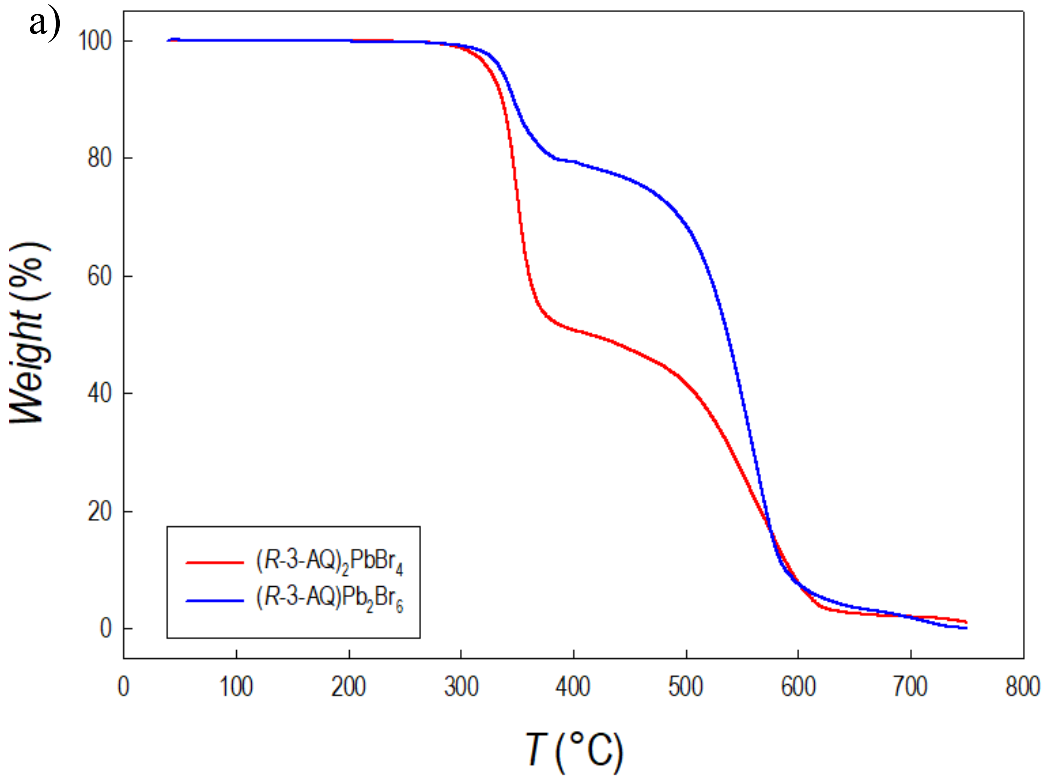

b)

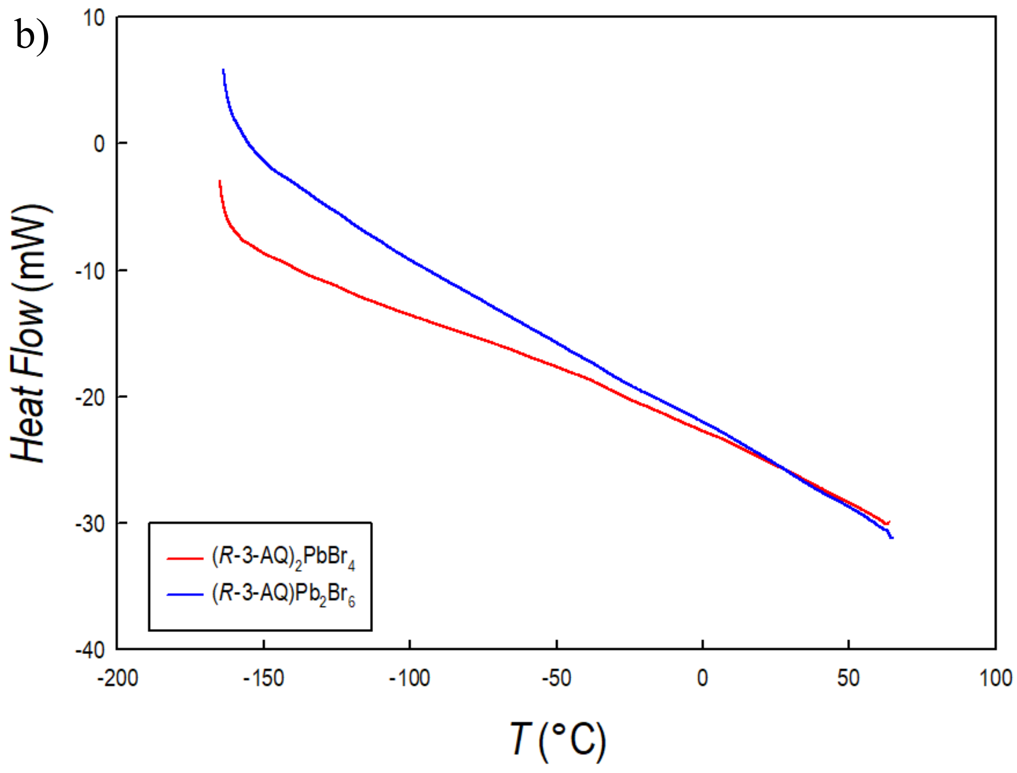

Figure S3. a) TGA curves for (R-3-AQ)$_2$PbBr$_4$·2Br (red line) and (R-3-AQ)Pb$_2$Br$_6$ (blue line); b) DSC curves for the same compounds.

a)

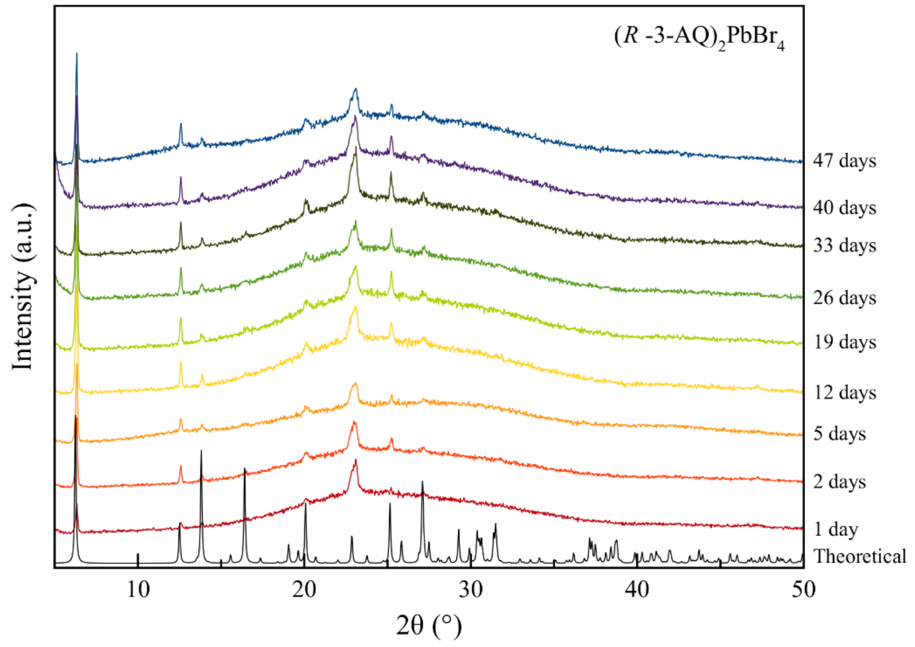

b)

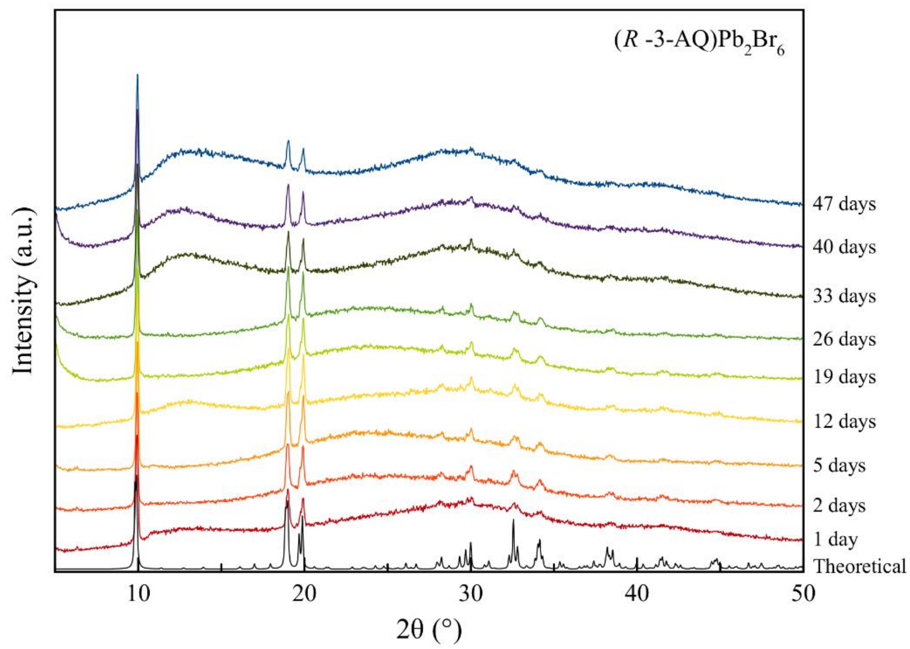

Figure S4. XRD patterns of a) (R-3-AQ)$_2$PbBr$_4$·2Br and (b) (R-3-AQ)Pb$_2$Br$_6$ as a function of time. Samples were left in the open air in the laboratory environment (T=22°C, RH=40%).

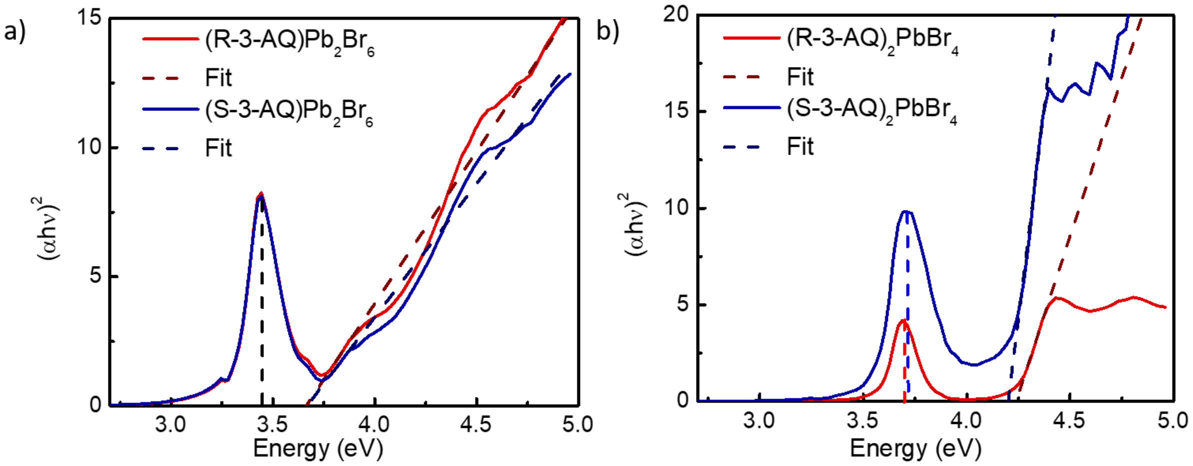

Figure S5. Tauc plot for (a) (R/S-3AQ)Pb$_2$Br$_6$ and (b) (R/S-3-AQ)$_2$PbBr$_4$·2Br at 77 K.

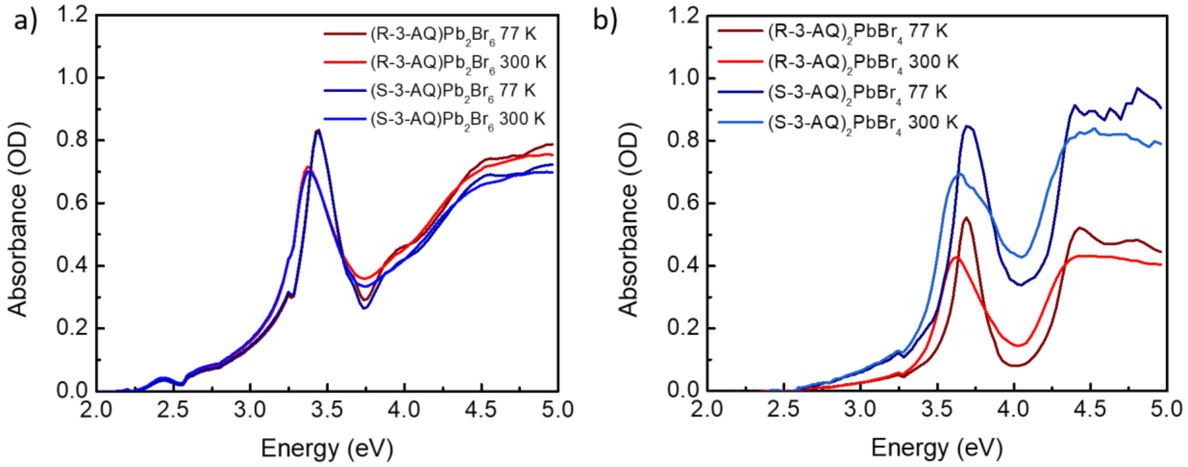

Figure S6. (a) Absorption spectra of (*R/S*-3-AQ)Pb_2Br_6 and (b) of (*R/S*-3-AQ)$_2PbBr_4$·2Br at 300 K and 77 K.

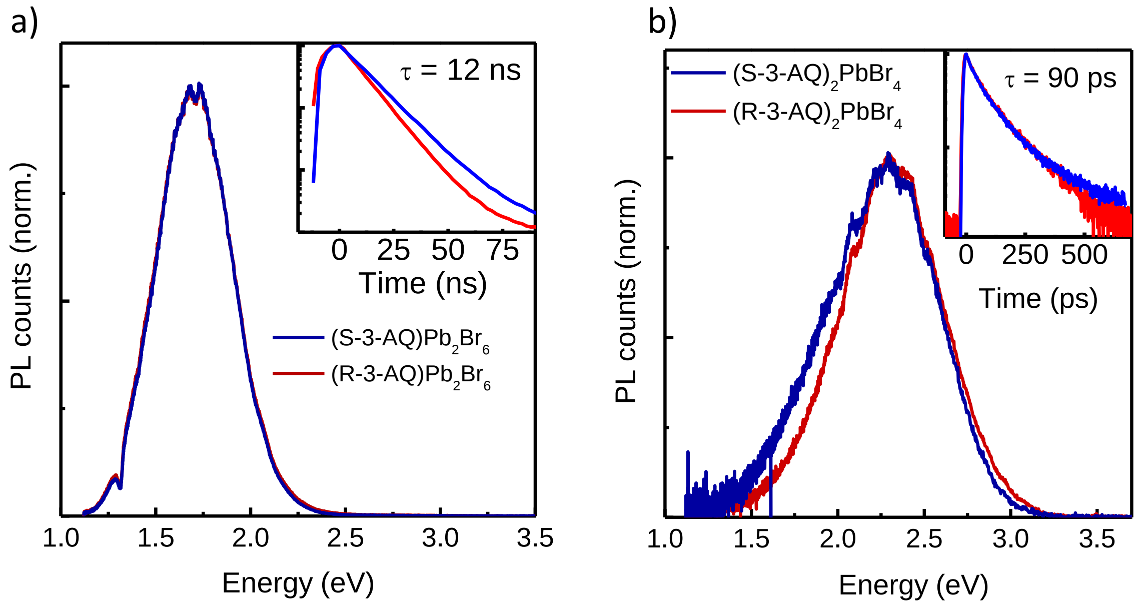

Figure S7. Photoluminescence spectra of, respectively, (*R*/*S*-3AQ)Pb_2Br_6 and (*R*/*S*-3-AQ)$_2PbBr_4$·2Br acquired at 300 K. In the inset, the room temperature PL lifetimes are shown.

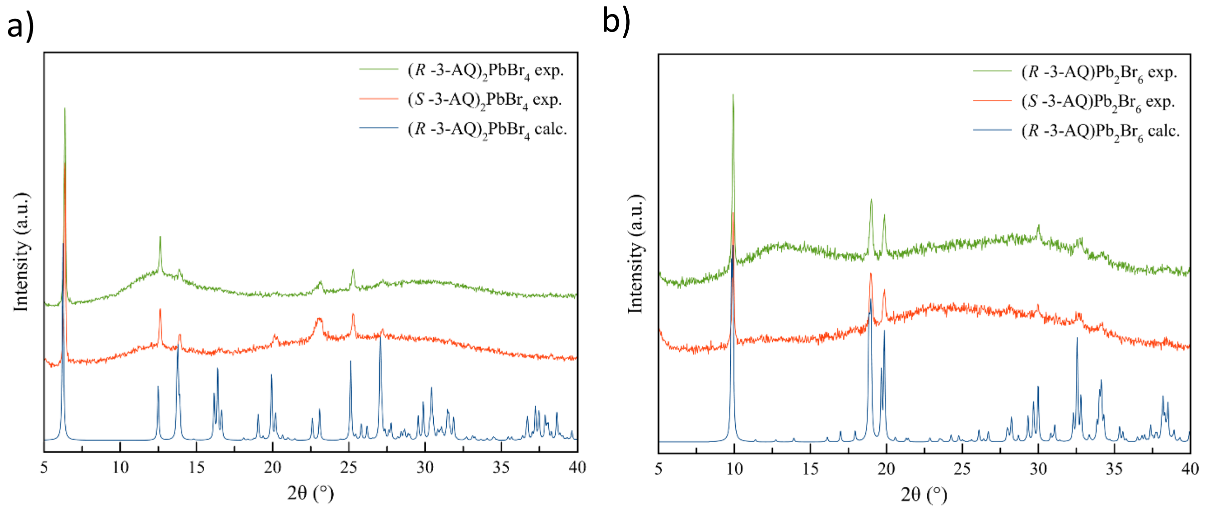

Figure S8. (a) XRD pattern of thin films of (a) (*R/S*-3-AQ)$_2$PbBr$_4$·2Br and (b) of (*R/S*-3-AQ)Pb$_2$Br$_6$ compared to the calculated pattern from single crystal data (blue pattern at the bottom).

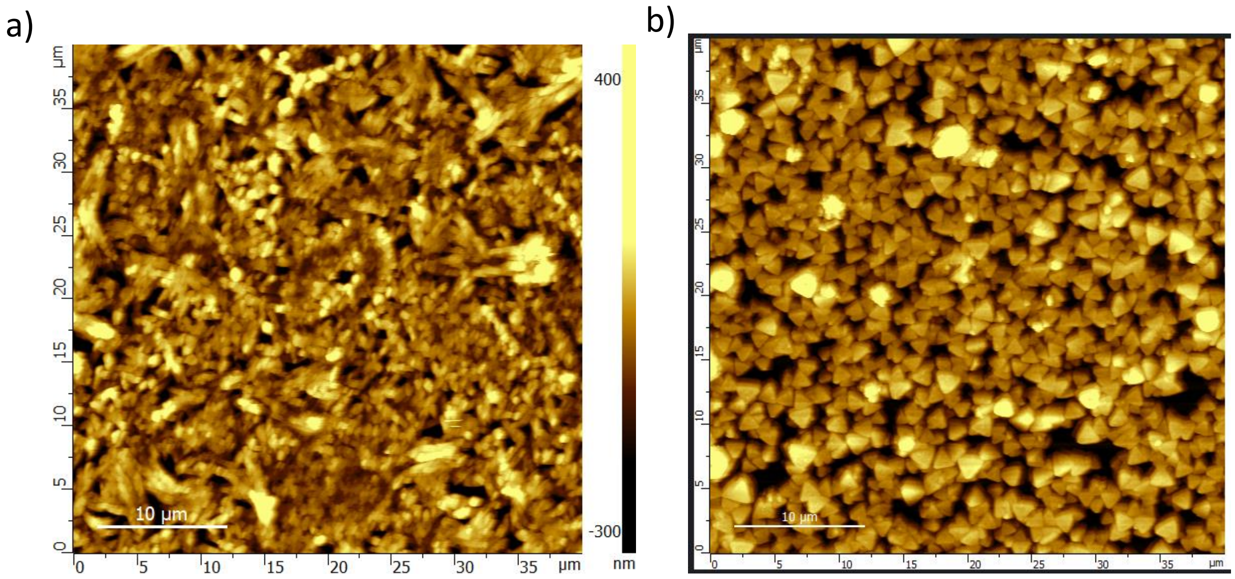

Figure S9. Representative AFM images of (a) (*R*-3-AQ)Pb$_2$Br$_6$ and (b) of (*R*-3-AQ)$_2$PbBr$_4$·2Br thin films.

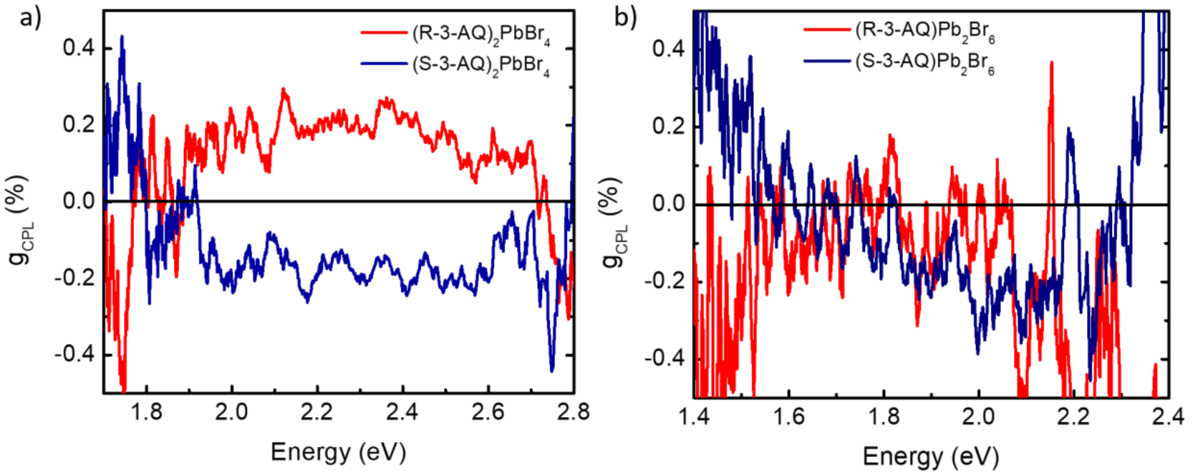

Figure S10. (a) CPL spectra of (a) (*R/S*-3-AQ)$_2$PbBr$_4$·2Br and (b) (*R/S*-3-AQ)Pb$_2$Br$_6$ at 77 K.

METHODS

Synthesis of (R/S-3AQ)Pb$_2$Br$_6$ and (R/S-3-AQ)$_2$PbBr$_4$·2Br.

To prepare (*R*/*S*-3AQ)Pb$_2$Br$_6$ and (*R/S*-3-AQ)$_2$PbBr$_4$·2Br, PbOAc$_2$ (Sigma-Aldrich 99.9%) was first dissolved in a large excess of 48% w/w aqueous HBr (Sigma-Aldrich) equivalent to 2 mL and the mixture was heated to 90°C. After the dissolution of the solid, the dihydrochloride salt of *R/S*-3-AQ (ApolloScientific, 97%, ee 98%) was added, with a diamine to metal molar ratio of 1:4 for (*R*/*S*-3AQ)Pb$_2$Br$_6$ and of 2:1 for (*R/S*-3-AQ)$_2$PbBr$_4$·2Br. To obtain the formation of single crystals the solution has been cooled down to room temperature at 1°C h^{-1}, then it was washed with anhydrous diethyl ether (Sigma-Aldrich 99.7%) and dried in vacuum at 65°C for 3 hours.

Thin films preparation. First, the glass substrates were washed using water, isopropanol and acetone in a sonicator for 20 min each and treated with oxygen plasma for 15 minutes. Then, the starting materials, prepared as indicated above, were dissolved in DMSO to obtain a 0.16 M final solution, and the thin films were prepared by spin coating method with the following program: 10 s, 150 rpm; 20 s, 350 rpm; 30 s, 1850 rpm. The films were annealed at 160°C for 2 min on a hot plate. Thin film thickness was determined by mechanical profilometer and resulted to be around 300 nm.

Single Crystal X-ray Diffraction. Measurements on selected crystal were collected on Rigaku XtaLab SuperNova (Rigaku Europe SE, Neu-Isenburg, Germany) four-circle diffractometer equipped by a

microfocus X-ray source (λ = 0.7107 Å) and a Dectris Pilatus 200K hybrid pixel array detector (DECTRIS AG, Baden-Daettwil, Switzerland). Data collection, data reduction and accurate measurements of the unit cell parameters were performed by means of the CrysAlisPro software suite, ver. 1.171.41.[1] Absorption effects were empirically evaluated based on a multi-scan approach using spherical harmonics by the SCALE3 ABSPACK scaling algorithm implemented in the CrysAlisPro crystallographic suite. Crystal structures were solved by direct methods (SIR 97) and refined by full-matrix least-square procedures on F^2 using all reflections (SHELXL 2018/3).[2,3]

Powder X-ray Diffraction. Powder X-ray diffraction (PXRD) data were acquired employing a Bruker D8 Advance θ:θ diffractometer and a Si zero-background sample-holder, in the 2θ range 3.0-105.0°, with steps of 0.02° and a time *per* step of 10 s and treated taking advantage of the TOPAS-6 software. Suitable unit cell parameters were retrieved upon indexing the PXRD pattern through the Singular Value Decomposition algorithm[4], then performing a whole powder pattern refinement with the Le Bail method[5]. The structure of (*S*-3AQ)Pb_2Br_6 was refined with the Rietveld method[6], generating the enantiomer by taking advantage of the atomic coordinates of (*R*-3AQ)Pb_2Br_6, solved by SC-XRD, and refining the atomic coordinates of the Pb and Br atoms. The background was described by means of a Chebyshev type polynomial function, and the instrumental contribution to the peak profile through the Fundamental Parameters Approach[7].

Atomic force microscopy (AFM). images were obtained with a SmartSPM microscope (Horiba Scientific) on the spin-coated thin films. Topographic imaging was performed in tapping mode with MikroMash NSC14 tips, with an amplitude of 120-140 nm and scan rate ranging from 1 to 0.5Hz.

UV-Vis spectroscopy. DRS spectra were acquired in the wavelength range 300- 800 nm directly on the powders by using a Jasco V-750 spectrophotometer, equipped with an integrating sphere (Jasco ISV-922).

Circular Dichroism. CD spectra were acquired in the wavelength range 350- 800 nm on thin films by using a Jasco J-1500 CD spectrophotometer. The g_{CD} value for both samples has been calculated after normalizing the absorptions using the following formula:

$$g_{cd} = \frac{\Delta A}{A}$$

where ΔA is the value obtained by dividing the mdeg by the constant (~32000) and A represents the absorption difference, calculated as:

$$A_L - A_R/_2$$

where A_L and A_R are the left and right absorptions.

The effect of Linear Dichroism (LD) was eliminated by performing measurements on the thin films tilted by 90° and then rotated by 180°.

Low Temperature absorbance: The materials absorbance at 77 K was measured in a spectrophotometer on thin films drop casted on fused silica substrates. The samples were mounted in a Linkam stage cryostat and cooled to 77 K with liquid nitrogen in vacuum condition. The excitonic energy level was identified as the central peak of a Gaussian feature, while the band gap energy was identified with a tauc fit.

Photoluminescence. Temperature-dependent photoluminescence was measured from bulk crystals under vacuum using a Linkam Stage cooled with liquid nitrogen. The sample was excited with CW laser (375 nm - TOPTICA) linearly polarized. PL was detected using a Maya2000 visible spectrometer. CD PL was acquired using with a quarter-wave plate set to ±45° relative to a linear polarizer. The samples were excited at a fluence of 100 mW/cm^2.

Time-resolved photoluminescence. For measurements with ns resolution, the 3d material was excited with the third harmonic (355 nm) from a Nd:YAG Picolo-AOT laser (pulse length of approximately 900 ps, 1 kHz repetition rate). An Andor spectrometer paired with an iStar iCCD is used for the detection. The camera acquisition is synchronized with the 1 kHz trigger signal from the laser. We track the photoemission dynamics by gating the camera's acquisition over a temporal window of 50 ns collected at increasing delay from the trigger reference (50 ns steps). The overall time resolution of the system is 2-4 ns. Kinetics are obtained by integrating the whole PL spectrum recorded at each delay. The samples were excited at a fluence of 100 mW/cm^2.

On the other hand to measure the shorter lifetime of the 2D material a shorter temporal resolution was required: we employed a Hamamatsu streak camera in Synchroscan mode, with a temporal resolution of 10 ps in the relevant measurement window (800 ps in total). The sample was excited at 350 nm using the second harmonic of a tunable Ti:Sapphire oscillator (Coherent Chameleon, 78 MHz, 150 ps).

Computational Details

Density functional theory (DFT) calculations were performed using the Quantum Espresso software package.[8] Structural optimizations for both two-dimensional (2D) and three-dimensional (3D) models were initiated from experimental CIF data. To account for van der Waals interactions, the Perdew–Burke–Ernzerhof (PBE) functional was employed with DFT-D3 dispersion corrections.[9] Norm-

conserving pseudopotentials were used for all structural optimizations, and electronic shells for Br (5s, 5p), N and C (2s, 2p), H (1s), and Pb (6s, 6p, 5d) were explicitly included.[10] The wavefunction cutoff and charge density cutoff were set at 60 Ry and 240 Ry, respectively. The Brillouin zone was sampled using a 2×2×2 k-point grid centered at the Gamma point for the 3D system and a 4×4×1 grid for the 2D system. Following structural relaxation, spin-orbit coupling (SOC) was introduced to accurately capture Rashba-type spin splitting, particularly significant for lead-containing systems. To refine electronic structure predictions and enhance the accuracy of band gap estimations, hybrid exchange-correlation functionals with varying fractions of exact exchange were subsequently applied. These results, presented in Table S2, highlight the impact of SOC and hybrid functional parameters on the electronic properties of both 2D and 3D configurations. PDOS analysis was carried out at HSE06-SOC level ($\alpha = 0.43$) using a 4×4×2 and 4×4×1 k-point grid centered at the Gamma point for the 3D system and for the 2D system, respectively, see Figure S12 and Figure 2 in the main text.

Table S3: Calculated band gap at different level of theory for the (*R*-3-AQ)Pb_2Br_6 and (*R*-3-AQ)$_2PbBr_4 \cdot 2Br$.

	(*R*-3-AQ)Pb_2Br_6	(*R*-3-AQ)$_2PbBr_4 \cdot 2Br$
PBE	2.81	3.01
PBE-SOC	2.06	2.38
HSE06-SOC α= 0.43	3.46	3.82
HSE06-SOC α= 0.25	2.84	3.19

Table S4: Calculated effective masses for different directions for the (*R*-3-AQ)Pb_2Br_6 compound at the PBE-D3 + SOC level of theory along with the Rashba data.

	m_e*	m_h*	μ		ε (meV)	Δk (x10^{-3} $Å^{-1}$)	α (eV Å)
Γ → X	0.75	1.01	0.43				
Γ → Y	0.34	0.54	0.21				
Γ → Z	0.53	0.66	0.29	VB	0.70	2.80	0.12
				CB	50.30	11.31	2.22
Γ → T	0.54	0.85	0.33				

Table S5: Calculated effective masses for different directions for the (*R*-3-AQ)$PbBr_4$ x 2Br compound at the PBE-D3 + SOC level of theory along with the Rashba data.

	m_e*	m_h*	μ		ε (meV)	Δk (x10^{-3} $Å^{-1}$)	α (eV Å)
R → A	0.55	1.14	0.37				
X → A	0.57	1.14	0.38				
Γ → M	0.42	0.55	0.24	VB	22.40	6.29	7.12
				CB	12.90	3.15	8.19
X → M	0.42	0.55	0.24				
Γ → Z	∞	∞	-				

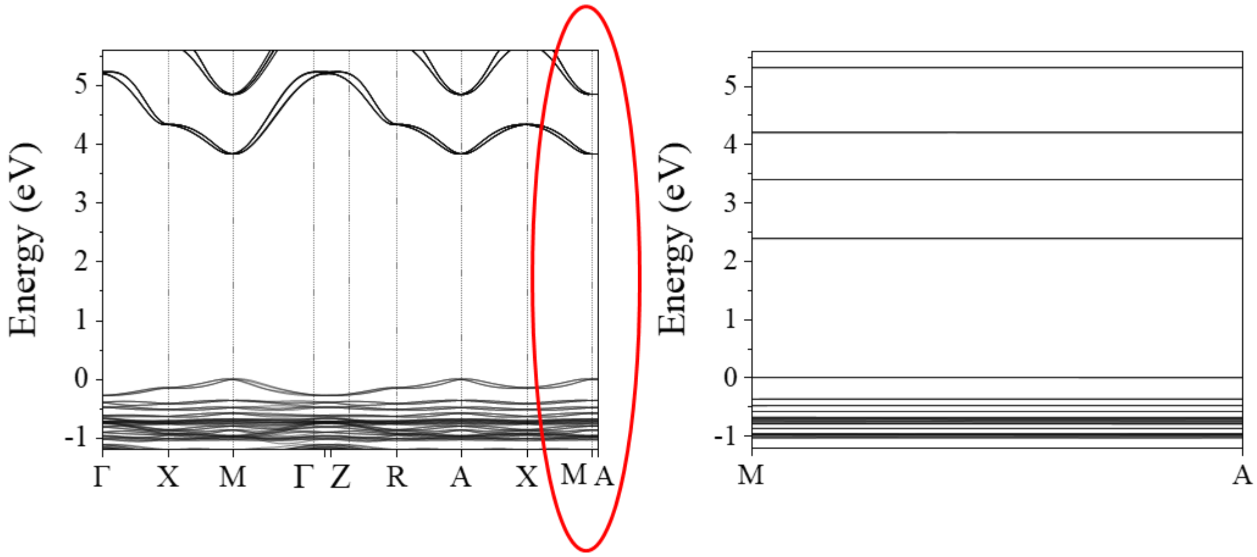

Figure S11: Close up on the flat Γ-> Z branch from the total band structure reported in the main text.

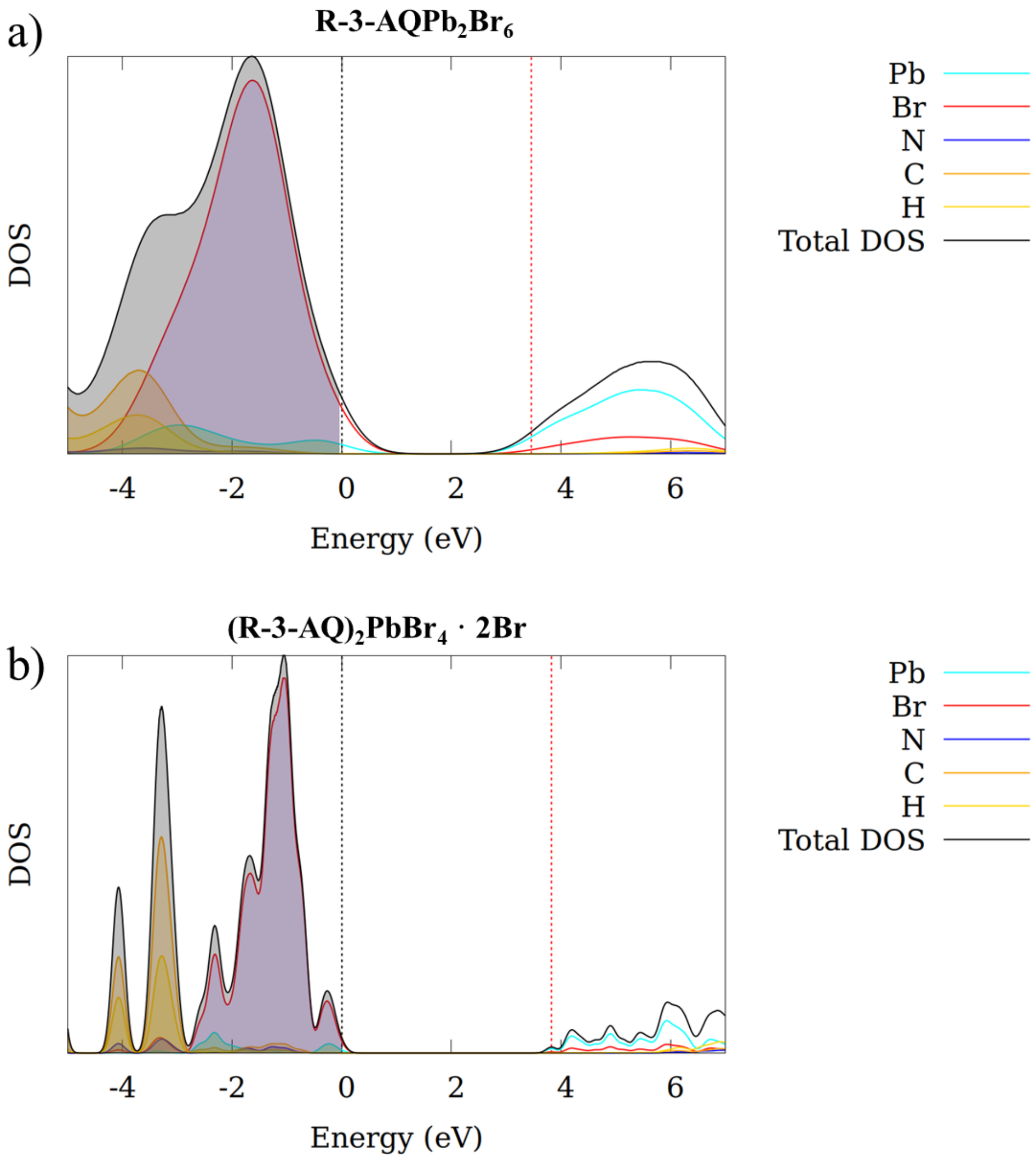

Figure S12: Projected density of states (PDOS) for the a) 3D and b) 2D materials, respectively. The PDOS was computed using the HSE06-SOC level of theory

References

(1) Rigaku Oxford Diffraction **2021**. CrysAlisPro Software system, version 1.171.41.104a, Rigaku Corporation, Wroclaw, Poland.

(2) Altomare, A.; Burla, M. C.; Camalli, M.; Cascarano, G. L.; Giacovazzo, C.; Guagliardi, A.; Moliterni, A. G. G.; Polidori, G.; Spagna, R. It SIR97: A New Tool for Crystal Structure Determination and

Refinement. *Journal of Applied Crystallography* **1999**, *32* (1), 115–119. https://doi.org/10.1107/S0021889898007717.
(3) Sheldrick, G. M. Crystal Structure Refinement with It SHELXL. *Acta Crystallographica Section C* **2015**, *71* (1), 3–8. https://doi.org/10.1107/S2053229614024218.
(4) Coelho, A. A. Indexing of Powder Diffraction Patterns by Iterative Use of Singular Value Decomposition. **2003**. https://doi.org/10.1107/S0021889802019878.
(5) Le Bail, A.; Duroy, H.; Fourquet, J. L. Ab-Initio Structure Determination of LiSbWO6 by X-Ray Powder Diffraction. *Materials Research Bulletin* **1988**, *23* (3), 447–452. https://doi.org/10.1107/S0021889802019878.
(6) Young, R. A. *The Rietveld Method*; IUCr Monograph on Crystallography-5; Oxford University Press, 1981.
(7) Cheary, R. W.; Coelho, A. A Fundamental Parameters Approach to X-Ray Line-Profile Fitting. *J Appl Crystallogr* **1992**, *25* (2), 109–121. https://doi.org/10.1107/S0021889891010804.
(8) Giannozzi, P.; Baroni, S.; Bonini, N.; Calandra, M.; Car, R.; Cavazzoni, C.; Ceresoli, D.; Chiarotti, G. L.; Cococcioni, M.; Dabo, I.; Dal Corso, A.; De Gironcoli, S.; Fabris, S.; Fratesi, G.; Gebauer, R.; Gerstmann, U.; Gougoussis, C.; Kokalj, A.; Lazzeri, M.; Martin-Samos, L.; Marzari, N.; Mauri, F.; Mazzarello, R.; Paolini, S.; Pasquarello, A.; Paulatto, L.; Sbraccia, C.; Scandolo, S.; Sclauzero, G.; Seitsonen, A. P.; Smogunov, A.; Umari, P.; Wentzcovitch, R. M. QUANTUM ESPRESSO: A Modular and Open-Source Software Project for Quantum Simulations of Materials. *J. Phys.: Condens. Matter* **2009**, *21* (39), 395502. https://doi.org/10.1088/0953-8984/21/39/395502.
(9) Grimme, S.; Antony, J.; Ehrlich, S.; Krieg, H. A Consistent and Accurate *Ab Initio* Parametrization of Density Functional Dispersion Correction (DFT-D) for the 94 Elements H-Pu. *The Journal of Chemical Physics* **2010**, *132* (15), 154104. https://doi.org/10.1063/1.3382344.
(10) Van Setten, M. J.; Giantomassi, M.; Bousquet, E.; Verstraete, M. J.; Hamann, D. R.; Gonze, X.; Rignanese, G.-M. The PseudoDojo: Training and Grading a 85 Element Optimized Norm-Conserving Pseudopotential Table. *Computer Physics Communications* **2018**, *226*, 39–54. https://doi.org/10.1016/j.cpc.2018.01.012.
